# One-Step Synthesis of Nitrogen-Doped TiO_2_ Heterojunctions and Their Visible Light Catalytic Applications

**DOI:** 10.3390/ma18102400

**Published:** 2025-05-21

**Authors:** Peng Lian, Aimiao Qin, Zhisen Liu, Hao Ma, Lei Liao, Kaiyou Zhang, Yingxi Qin

**Affiliations:** 1 Key Laboratory New Processing Technology for Nonferrous Metals & Materials Ministry of Education, Guangxi Key Laboratory of Optical and Electronic Materials and Devices, Guangxi Key Laboratory of Environmental Pollution Control Theory and Technology, College of Materials Science & Engineering, Guilin University of Technology, Guilin 541004, China; lianpeng@gdupt.edu.cn (P.L.); 1995004@glut.edu.cn (L.L.); kaiyou2014@glut.edu.cn (K.Z.); qinyingxi@dicp.ac.cn (Y.Q.); 2 College of Chemistry, Guangdong University of Petrochemical Technology, Maoming 525000, China; lzs_415@163.com (Z.L.); thma@gdupt.edu.cn (H.M.)

**Keywords:** dopant, heterostructure, photochemical, hierarchical, semiconducting

## Abstract

In this study, nitrogen-doped TiO_2_ heterojunction materials were successfully synthesized via a facile one-step solvothermal approach. A range of advanced characterization techniques were employed to thoroughly analyze the structural and compositional properties of the synthesized photocatalysts, and their application potential for tetracycline (TC) degradation under visible light was studied. The results indicated that N-doped TiO_2_ exhibited a well-defined hierarchical micro/nanostructure and formed an efficient anatase/rutile homogeneous heterojunction. The photocatalytic performance of N-TiO_2_ for TC degradation under visible light was significantly enhanced, achieving a degradation efficiency of up to 87% after 60 min of irradiation. This improvement could be attributed to the synergistic effects of optimal nitrogen doping, heterojunction formation, and the hierarchical micro/nanostructure, which collectively reduced the bandgap energy and suppressed the recombination rate of photogenerated carriers. Furthermore, density functional theory (DFT) calculations were conducted to systematically explore the impacts of substitutional and interstitial nitrogen doping on the energy band structure of TiO_2_.

## 1. Introduction

The issue of environmental pollution is becoming increasingly severe with the advancement of society and the rapid growth of the contemporary chemical industry. In particular, water pollution has emerged as a critical concern [1,2]. In recent decades, the extensive use of antibiotics has led to a significant increase in the presence of toxic and persistent organic pollutants in wastewater, including tetracycline (TC) wastewater [3,4,5]. TC wastewater is characterized by its diverse chemical composition, varying water quality characteristics, and pronounced ecotoxicological effects. Additionally, its discharge significantly promotes the growth of antibiotic-resistant bacteria and facilitates the spread of antibiotic resistance genes. Consequently, addressing the treatment of TC wastewater has emerged as an urgent societal concern. Currently, wastewater treatment methods are primarily categorized into physical, biological, and chemical approaches [6]. Although the physical method is simple to operate, its efficiency is low, and it can easily lead to secondary pollution [7]. On the other hand, the biological method requires advanced equipment and precise reaction conditions, which pose significant challenges in practical implementation [8]. The chemical treatment of sewage is highly efficient and minimizes the risk of secondary pollution, particularly the photocatalytic oxidation method, which effectively converts large molecular pollutants into environmentally friendly small molecules [9,10]. Consequently, this approach holds significant promise for widespread application and has emerged as a prominent area of research both domestically and internationally.

Since the discovery of the photocatalytic effect of semiconductor materials in the 20th century, research on semiconductor photocatalysts has witnessed a significant surge, particularly due to their pivotal role in organic matter degradation within wastewater [11]. The TiO_2_ stands out as the optimal photocatalyst due to its exceptional catalytic activity, robust chemical stability, and non-toxic nature [12,13,14]. In recent decades, a great deal of research has been conducted on TiO_2_ in order to address the limitations of single-component TiO_2_, such as low degradation efficiency of heterogeneous system pollutants, poor response to visible light, and facile recombination of photogenerated carriers [15,16,17]. These efforts include doping [8,18], morphology design [19,20], or combining with other semiconductors to form semiconductor heterojunction [21]. Doping is a prevalent strategy employed to enhance the catalytic performance of TiO_2_. The doping of non-metallic elements (such as N [22,23,24], C [25], S [26], and P [27]) into TiO_2_ has been demonstrated to exhibit a significant enhancement in visible light absorption capacity. The sol-gel method was employed by Sun et al. [28] to synthesize amorphous titanium dioxide doped with N atoms. The photocatalytic degradation rate of samples for TC solution reached 92.23% after 60 min of visible light irradiation. They believed that an excess of N sources could potentially result in the coverage or blockage of active sites on TiO_2_, which lead to a decline in photocatalytic activity. Currently, it is widely acknowledged that an optimal nitrogen concentration promotes the enhancement of photocatalytic performance. However, excessive doping of nitrogen can have a detrimental effect on the position of the valence band (<1.23 V vs. the reversible hydrogen electrode, RHE), leading to a reduction in photocatalytic efficiency [29]. Hierarchical structured materials are micro/nanostructural systems composed of multiple low-dimensional basic units, where the overall dimensionality exceeds that of each constituent unit. Specifically, these materials consist of nano-scale building blocks with varying dimensions, morphologies, and compositions. Constructing TiO_2_ with hierarchical micro/nanostructures is an effective strategy to overcome its inherent shortcomings. For example, designing a hierarchically structured porous network with interconnections can promote the migration of reactants to the active sites on the pore walls. Reducing the basic unit size of TiO_2_ catalyst materials to the nanometer level can not only introduce the quantum size effect but also effectively regulate the conduction band and valence band positions of its structure, thereby achieving performance optimization [30]. Furthermore, despite the extensive research on N-doped TiO_2_, reports on the synthesis of hierarchical micro/nanostructure TiO_2_ heterojunction materials via a one-step low-temperature method remain relatively scarce. Therefore, the investigation of N-doped TiO_2_ continues to hold substantial research value within the academic domain.

Herein, we report a facile one-step approach for the synthesis of N-doped TiO_2_ under mild reaction conditions, devoid of any acid or base. By varying the N/Ti molar ratio, various hierarchical micro/nanostructure N-TiO_2_ heterojunctions were prepared via the solvothermal. Moreover, these samples exhibited significant TC photodegradation under visible light, attributed to the phase composition, enhanced optoelectrical properties, and favorable morphology. Density functional theory (DFT) was employed to investigate the alterations in the band gap energy of TiO_2_ caused by N doped at substitutional and interstitial sites. Based on the results, the mechanism by which N-doped anatase TiO_2_ exhibited higher photocatalytic activity in the visible light region compared with pure TiO_2_ was elucidated.

## 2. Experimental

### 2.1. Materials

Cetyltrimethylammonium bromide (C_19_H_42_BrN, CTAB), ethanol (C_2_H_6_O), tetrabutyl titanate (C_16_H_36_O_4_Ti, TBOT), Tetracycline (C_22_H_24_N_2_O_8_), Urea (H_2_NCONH_2_), and commercial TiO_2_ (P25) were obtained from Macklin Biochemical Co., Ltd. (Shanghai, China). All these chemical reagents were obtained as received, without requiring further purification.

### 2.2. Synthesis of N-TiO_2_

The N-TiO_2_ was synthesized via a one-step solvothermal method at low temperature, with the morphology and photocatalytic degradation of TiO_2_ being modulated by adjusting the molar ratios of N/Ti. In a typical process, 0.72 g CTAB was dispersed in 50 mL ethanol and maintained, stirring constantly, for 30 min; next, 6 mL of TBOT was slowly added through the pipette, and the addition of urea as a nitrogen source was based on the molar ratios of N/Ti at concentrations of 0.05 M. The mixed solution was stirred for 1 h and then transferred into a Teflon-lined stainless steel autoclave with a capacity of 100 mL. Subsequently, the autoclave was heated at 100 °C for 24 h and then cooled down to an ambient temperature naturally. Next, the as-obtained product was centrifuged, washed, and re-dispersed in water and ethanol for three cycles. After that, the product was dried at 70 °C in an oven for 24 h and then calcined at 500 °C in the air for 2 h. Finally, the product was labeled as N-TiO_2_-0.05. For comparison, N-TiO_2_-0.025 and N-TiO_2_-0.1 were also prepared using the same procedures with molar ratios of N/Ti being 0.025 M and 0.1 M, respectively. Likewise, the solvothermal synthesis material without the addition of urea was referred to as the reference TiO_2_ sample.

The synthesis mechanism can be described as follows: butyl titanate (Ti(OBu)_4_) generated TiO_2_ precursors via hydrolysis and polycondensation reactions in an ethanol medium. Specifically, butyl titanate initially underwent a hydrolysis reaction in absolute ethanol to produce titanium hydroxyl compounds (Ti-OH). Subsequently, these compounds further participated in polycondensation reactions to form Ti-O-Ti bonds, ultimately leading to the formation of TiO_2_. When the concentration of the surfactant surpassed its critical micelle concentration (CMC), micelles spontaneously assembled. These micellar structures served as templates that guided the deposition of TiO_2_ precursors at their interfaces, thereby promoting the formation of hierarchical nanosphere TiO_2_. The reaction equations are as follows:

$${Ti\left(OBu\right)}_{4}+{xH}_{2}O={Ti\left(OH\right)}_{x}{\left(OBu\right)}_{4-x}+xBuOH$$

$$2{Ti\left(OH\right)}_{4}={Ti}_{2}{O\left(OH\right)}_{6}+H_{2}O$$

$${Ti}_{2}{O\left(OH\right)}_{6}=2TiO_{2}+3H_{2}O$$

Urea (CO(NH_2_)_2_) decomposed under high-temperature conditions, such as calcination or hydrothermal synthesis, releasing nitrogen-containing species, including NH_3_ and CN^−^. Notably, the released NH_3_ could serve as a nitrogen source and react with the TiO_2_ precursor. This reaction enabled some N atoms to incorporate into the interstitial sites of the TiO_2_ lattice, forming a stable Ti-O-N structure. The relevant reaction equations are as follows:

$$C{O\left({NH}_{2}\right)}_{2}={NH}_{3}+HNCO$$

$$2TiO_{2}+2x{NH}_{3}=2Ti{O_{2-x}N}_{x}+{3xH}_{2}O$$

### 2.3. Sample Characterization

The crystalline structures were characterized using an X-ray diffraction (XRD) analysis (Ul-tima IV, Rigaku, Tokyo, Japan) with Cu Kα radiation at a scanning rate of 10°/min in the 2θ range of 10–80°. FESEM and EDS analyses were performed using an S-4800 (Hitachi, Tokyo, Japan) equipped system. TEM measurements were conducted on a JEM-2010HR (JEOL, Tokyo, Japan). N_2_ adsorption studies were carried out using an ASAP 2010 analyzer (Micromeritics, Norcross, GA, USA). The pore size distribution curves were derived from the desorption branch, and the specific surface areas were calculated using the Brunauer–Emmett–Teller (BET) model. Fourier transform infrared spectroscopy (FT-IR) analysis (Nicolet 6700, Thermo Scientific, Waltham, MA, USA) was performed over the wavenumber range of 500–4000 cm^−1^ with a resolution of 4 cm^−1^ and a transmittance scan mode. Raman spectra were recorded using a DXR 2 (Thermo Scientific) with a 785 nm He-Ne laser, covering the wave number range of 50–1000 cm^−1^. The surface characterization of the sample was performed using X-ray photoelectron spectroscopy (XPS) (ESCALAB 250Xi, Thermo Scientific). Electron paramagnetic resonance (EPR) spectra were recorded at room temperature using a Bruker EMXplus (Berlin, Germany) system. The UV-Vis diffuse reflectance spectrum (UV-Vis DRS) was obtained using a UV-270 spectrometer (Kyoto, Japan). Photoluminescence (PL) spectra were recorded using an FLS980 spectrophotometer (Edinburgh, UK), with an excitation wavelength of 320 nm and a 400 V test voltage. Intermediate compounds formed during TC degradation were identified using a liquid chromatograph-mass spectrometer (HPLC-MS) system (Thermo Scientific, USA). Transient photocurrent (TPC) measurements were performed using an Electrochemical Station (Shanghai Chenhua, CHI 660C, Shanghai, China), employing a three-electrode system with 0.01 M Na_2_SO_4_ electrolyte and a 300W Xe lamp (λ > 300 nm).

### 2.4. Photocatalytic Degradation of TC

To establish the adsorption–desorption equilibrium prior to photoirradiation, 50 mg of the as-prepared photocatalyst was dispersed into 100 mL of a 20 mg L^−1^ aqueous TC solution. The suspension was magnetically stirred in the dark for 30 min at room temperature. An ozone-free xenon arc lamp (HXF 300W, 300 watts) equipped with an ultraviolet cut-off filter (λ > 420 nm) was utilized as the visible light source, with a lamp intensity of 1500 mW/cm^2^. Upon achieving adsorption equilibrium, the photocatalytic degradation experiment was initiated by exposing the system to Xe lamp irradiation. At specified time intervals, 3 mL aliquots of the reaction suspension were collected. The samples were then filtered through a 0.22 μm polytetrafluoroethylene (PTFE) membrane syringe filter to remove catalyst particles. The concentration of TC in the filtrate was subsequently analyzed using a UV-vis spectrophotometer at its characteristic absorption wavelength of 360 nm.

## 3. Results and Discussion

**Figure 1:**
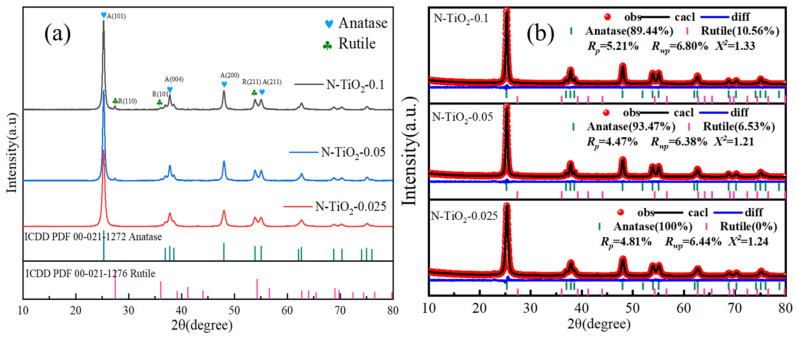
(**a**) XRD pattern of samples. (**b**) Refined XRD pattern of samples.

The crystal structures of N-TiO_2_ samples were systematically investigated using XRD analysis, as presented in Figure 1. The XRD patterns clearly demonstrated that all the observed diffraction peaks could be attributed to tetragonal anatase (ICDD PDF 00-021-1272) and rutile (ICDD PDF 00-021-1276) TiO_2_, confirming the high purity of the prepared photocatalysts. The X-ray diffraction patterns of TiO_2_ included peaks at 2θ values of 25.3°, 37.8°, 48.0°, and 55.1°, corresponding to the (101), (004), (200), and (211) planes of anatase, respectively. Additionally, the peaks observed at 2θ angles of 27.4°, 36.1°, and 54.3° were attributed to the (110), (101), and (211) planes of rutile [31]. The refined XRD patterns presented in Figure 1b demonstrate that as the nitrogen content increased, the proportion of the rutile phase rose significantly, although the anatase phase remained dominant. Specifically, the ratios of the anatase/rutile phases were 93.47/6.53 for the N-TiO_2_-0.05 sample and 89.44:10.56 for the N-TiO_2_-0.1 sample. Furthermore, with the increase in nitrogen doping concentration, the cell parameters exhibited a decreasing trend. This phenomenon could be attributed to nitrogen atoms being incorporated into the titanium dioxide lattice as interstitial impurities. The limited space available for these interstitial positions led to a compressive effect on surrounding atoms, consequently reducing the cell parameters. Specific values are detailed in Table S1. Therefore, the peaks of A (101) and R (110) exhibited a slight shift toward higher angles, indicating that the dopants were incorporated into the TiO_2_ matrix [32], as shown in Figure S1.

Furthermore, the crystallite size was calculated using the Debye–Scherrer equation, as detailed below:

(1) $$D=\frac{k\lambda }{\beta cos\theta }$$

The crystallite size (D) of the N-TiO_2_ samples was calculated using the Scherrer equation, wherein the Scherrer constant (*K* = 0.89) was applied along with the X-ray wavelength (*λ* = 1.54 Å for Cu Kα radiation) and the full width at half maximum (FWHM) of the diffraction peaks. The crystallite sizes of the N-TiO_2_-0.025, N-TiO_2_-0.05, and N-TiO_2_-0.1 samples were determined based on the principal reflections, yielding average crystallite diameters of 12.4 nm, 14.6 nm, and 20.4 nm, respectively. These results demonstrated that the crystallite size of N-TiO_2_ nanoparticles increased with the increase in the N content. According to the report by Japa [33], the coexistence of anatase and rutile crystalline phases was found to be beneficial for achieving enhanced photocatalytic activity. This phenomenon could be primarily attributed to the distinct properties of TiO_2_ phases. Anatase TiO_2_ exhibited superior photocatalytic activity due to its strong adsorption tendencies for organic compounds and reduced electron-hole recombination rates. In contrast, rutile TiO_2_, characterized by a narrower band gap (3.0 eV), could be efficiently excited by light at 410 nm and functioned as an electron sink because of its lower band gap compared with anatase TiO_2_ (3.2 eV). Consequently, it was anticipated that the phase-separated structure in these samples likely contributed to enhanced photocatalytic activity through the formation of a heterojunction.

**Figure 2:**
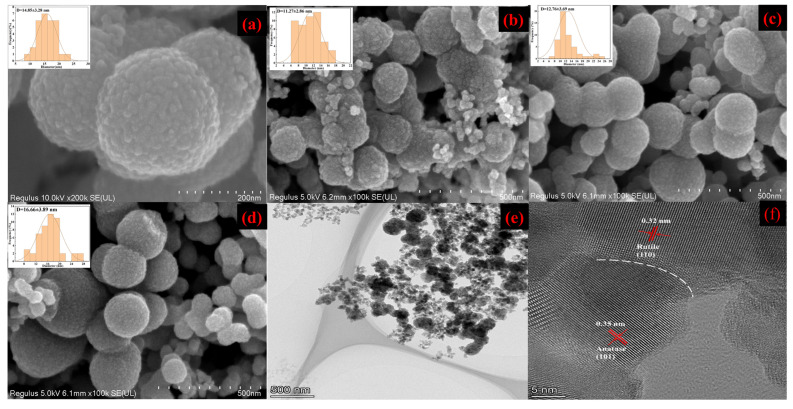
SEM images and surface nanoparticle diameter distribution (inset) of (**a**) pure TiO_2_, (**b**) N-TiO_2_-0.025, (**c**) N-TiO_2_-0.05, and (**d**) N-TiO_2_-0.1 and (**e**) TEM and (**f**) HRTEM images of N-TiO_2_-0.05.

The SEM images of the pure TiO_2_ and N-TiO_2_-X nanocomposites, as depicted in Figure 2, clearly revealed the spherical morphology of the synthesized material with a rough texture. The surface was composed of numerous nanoparticles, and the diameter distribution of these nanoparticles ranged from 11 to 22 nm. Additionally, N doping had limited influence on the micro/nano hierarchical structure of TiO_2_. Notably, the diameter of the spherical particles increased as the N/Ti molar ratio rose from 0.025 M to 0.1 M, and the size of the surface nanoparticles also expanded within the observed range. These findings were in agreement with theoretical predictions derived from the Scherrer formula. The diameters of N-TiO_2_-0.025, N-TiO_2_-0.5, and N-TiO_2_-0.1 were about 200 nm (Figure 2b), 220 nm (Figure 2c), and 250 nm (Figure 2d), respectively. This indicated the size of N-TiO_2_ could be controlled by adjusting the molar ratios of N/Ti. The special hierarchical micro/nanostructures of N-TiO_2_ were expected to enhance the photocatalytic activity, owing to the efficient separation of photogenerated electron-hole pairs.

To confirm the presence of nitrogen dopants in TiO_2_, EDS analysis was used to determine the elemental composition of N-TiO_2_-0.05. The elemental mapping and EDS spectra (Figure S2) of the N-TiO_2_-0.05 sample confirmed the existence of the nitrogen elements within the TiO_2_, which was consistent with XRD results.

As shown in Figure 2e,f, high-resolution transmission electron microscopy (HRTEM) analysis offered a comprehensive understanding of the nanostructural characteristics of N-TiO_2_-0.05, with its atomic-scale lattice fringes being clearly resolved. The morphology of N-TiO_2_-0.05 can be observed in Figure 2e, revealing a solid spherical structure characterized by a remarkably uniform size. The HRTEM image in Figure 2f reveals distinct and well-defined lattice stripes of N-TiO_2_-0.05. More importantly, the HRTEM image clearly revealed the coexistence of lattice spacings measuring 0.32 nm and 0.35 nm, which corresponded to the (110) plane of the rutile phase and the (101) plane of the anatase phase, respectively [33,34]. Therefore, the HRTEM observation further corroborated the presence of the junction phase, thereby supporting the findings from XRD spectroscopy.

**Figure 3:**
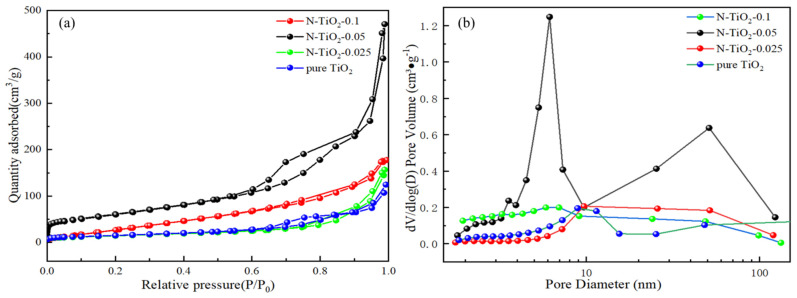
(**a**) N_2_ adsorption–desorption isotherm and (**b**) the pore size distribution curves of the synthesized samples.

The specific surface area and corresponding pore size distribution of the synthesized N-TiO_2_-X nanomaterials were determined through adsorption–desorption isotherm analysis of the N_2_ isotherm data presented in Figure 3. According to the IUPAC classification, TiO_2_ and N-TiO_2_-0.05 samples exhibited type IV isotherms with an H3 hysteresis loop [35], indicating the inherent ordered mesoporous nature, and consisted of large macropores in both the samples (Figure 3a). The adsorption isotherms of samples N-TiO_2_-0.025 and N-TiO_2_-0.1 were classified as II type isotherms according to their characteristics. The BET surface area analysis revealed a significant enhancement in the BET surface area of TiO_2_ upon N doping, increasing from 57 cm^3^/g (reference TiO_2_) to 218 cm^3^/g (N-TiO_2_-0.05), which was 3.8 times the reference TiO_2_. The specific surface area, pore volume values, and pore size were determined by employing the multi-point BET method in conjunction with the Barrett–Joyner–Halenda (BJH) model (Table S2). As depicted in Figure 3b, nitrogen atom doping significantly altered the pore volume and pore size within TiO_2_. Specifically, with the increase in the N doping amount, the pore volume exhibited a trend of initially rising and then declining, while the pore size progressively diminished. This may be attributed to the fact that when the doping level of N was relatively low, it could influence the transformation of the colloid structure during the synthesis of the TiO_2_ precursor, thereby facilitating the formation of mesopores and significantly enhancing the material’s porosity. However, excessive N doping may result in the blockage of pore channels, consequently reducing the porosity [36].

**Figure 4:**
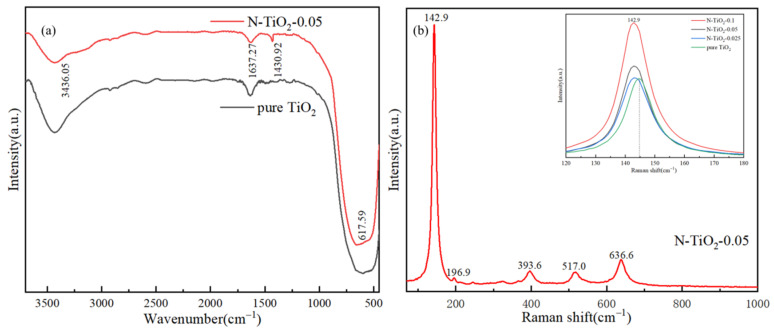
(**a**) The FT-IR spectra of samples. (**b**) Raman spectra of samples.

The FT-IR spectra of TiO_2_ and N-TiO_2_-0.05 are presented in Figure 4a. The peak at 3436 cm^−1^ observed in both N-TiO_2_ and TiO_2_ was attributed to the stretching vibration of O-H groups adsorbed on their surfaces. Additionally, the peak at 1637 cm^−1^ corresponded to the bending vibration of H-O-H groups associated with water molecules adsorbed on the photocatalyst surface. The adsorption of hydroxyl groups on photocatalyst surfaces has been extensively acknowledged for its critical role in enhancing photocatalytic activity. This enhancement is primarily due to the transfer of photogenerated species facilitated by these hydroxyl groups during photocatalytic reactions, which promotes the generation of reactive species and effectively reduces the recombination rate of electron-hole pairs. Moreover, the broad absorption bands in the 500–800 cm^−1^ range could be ascribed to the Ti-O-Ti stretching vibrations present in both N-TiO_2_ and TiO_2_. Furthermore, the peak at 1430 cm^−1^ was observed in the N-TiO_2_-0.05 sample, which could be attributed to the N-H bending vibration, likely originating from by-products such as NH₄^+^ formed during urea decomposition [37].

The crystalline structure of N-TiO_2_-0.05 was further characterized by Raman spectroscopy (Figure 4b). The Raman peaks observed at 142.9, 196.9, 393.6, 517.0, and 636.6 cm⁻¹ within the 150–1000 cm^−1^ spectral region corresponded to the characteristic vibration modes of anatase TiO_2_. The prominent presence of these anatase-specific peaks confirmed the successful formation of a highly crystalline anatase phase in N-TiO_2_-0.05, which was in excellent agreement with the XRD analysis results. Among them, 143, 393, and 517 cm^−1^ peaks corresponded to the E_g_, B_1g_, and A_1g_ + B_1g_ modes, respectively. Furthermore, as depicted in Figure 4b (inset), the augmentation of the molar ratios of N/Ti induced a blue shift (from 144.9 cm^−1^ to 142.9 cm^−1^) and broadening of the E_g_ mode peak in N-TiO_2_-X, which could be attributed to the generation of Ti^3+^ species and oxygen vacancies within N-TiO_2_-X. The blue shift of the Raman peak could be attributed to the difference in electronegativity between nitrogen and oxygen. Post-doping, the bond energy and length of the Ti-N bond exhibited significant differences compared with those of the Ti-O bond, characterized by a stronger bond energy and shorter bond length. Based on the principles of Raman scattering, an increase in bond strength resulted in higher vibration frequencies, leading to the observed blue shift in the Raman peak. The broadening of the E_g_ peak could be attributed to the presence of a mixed phase of anatase and rutile in nitrogen-doped TiO_2_. This phenomenon resulted from the superposition of vibration modes originating from the distinct phases, leading to the observed broadening of the Raman peak. Raman spectroscopy results demonstrated that nitrogen doping promoted the formation of oxygen vacancies, thereby potentially enhancing the photoelectrochemical activity.

**Figure 5:**
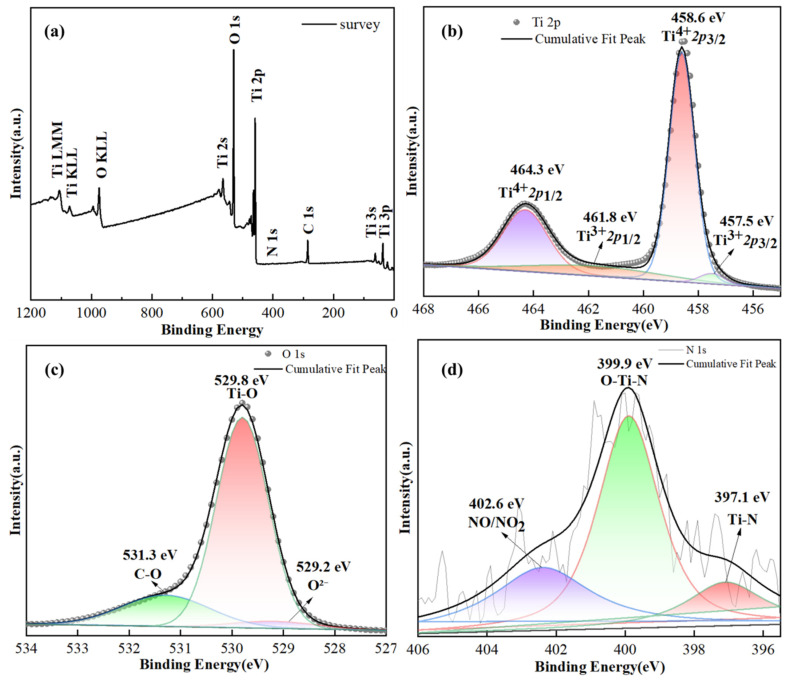
XPS spectra of N-TiO_2_-0.05. (**a**) Survey, (**b**) Ti 2p, (**c**) O 1s, and (**d**) N 1s.

The distribution and valence state of elements on the surface of N-TiO_2_-0.05 were investigated using XPS technology, as illustrated in Figure 5. The XPS survey and high-resolution XPS spectra of the N-TiO_2_-0.05 sample for three elements (Ti, O, and N) are presented in Figure 5. The XPS survey (Figure 5a) revealed that the composition of the structure primarily consisted of C, Ti, O, and N elements. The presence of a carbon C 1s peak at 285 eV may come from CO_2_ or C pollution adsorbed in the air [38].

The XPS spectra of Ti 2p for the N-TiO_2_-0.05 sample (Figure 5b) revealed that the peaks at 458.6 eV and 464.3 eV were assigned to the Ti^4^⁺ 2p₃/_2_ and Ti^4^⁺ 2p_1_/_2_ orbitals in TiO_2_, respectively. The binding energy difference of 5.7 eV between these two peaks aligned with the characteristic Ti^4^⁺-O bond in TiO_2_. Furthermore, the peaks at 457.7 eV and 461.8 eV were attributed to the Ti^3^⁺ 2p_3_/_2_ and Ti^3^⁺ 2p_1_/_2_ orbitals, respectively [39]. N doping can potentially lead to the reduction of Ti^4^⁺ to Ti^3^⁺ via the charge compensation mechanism. Specifically, N atoms possess a different outer electron configuration compared with O atoms. When N atoms substitute for O atoms in the titanium dioxide lattice, their lower electronegativity causes N to donate electrons. To preserve the electrical neutrality of the crystal, neighboring Ti^4^⁺ ions accept these electrons and are consequently reduced to Ti^3^⁺. The presence of the Ti^3+^ chemical state and a slightly lower binding energy (BE) of Ti^3+^ 2p_1/2_ and Ti^3+^ 2p_3/2_, compared with those reported in the literature, could be attributed to substitutional doping of the TiO_2_ lattice with nitrogen ions [40,41]. In the O 1s XPS spectrum (Figure 5c), three distinct peaks were observed at binding energies of 529.2, 529.9, and 531.6 eV. These peaks were attributed to lattice oxygen within the TiO_2_ matrix, oxygen atoms involved in Ti-O chemical bonds, and non-lattice oxygen species such as surface hydroxyl groups. According to the findings [42], the O 1s peak of the P25 nanoparticle was observed at 530.8 eV, which underwent a shift toward lower binding energy (530.0 eV) in N-doped TiO_2_ NP. This study also revealed similar peak shifts compared with those reported [40,42]. The N 1s spectrum in Figure 5d exhibited three peaks at 397.1, 399.9, and 402.6 eV [43]. The distinct absorption peak at 399.9 eV clearly indicated the presence of nitrogen atoms within the O-Ti-N environment. Peaks located at 397 and 402.4 eV further characterized the nitrogen species, specifically substitutional nitrogen in the Ti-N structure and nitrogen in oxidized states (e.g., NO or NO_2_) within the N-TiO_2_-0.05 sample [36].

**Figure 6:**
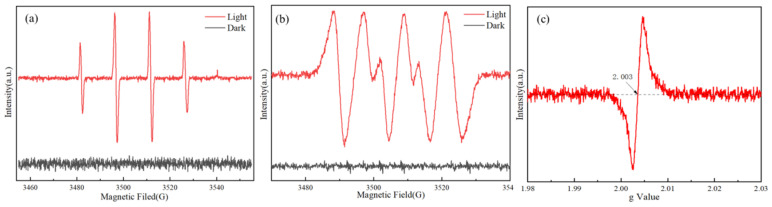
The EPR spectra of N-TiO_2_-0.05: (**a**) hydroxyl radical, (**b**) superoxide radical, and (**c**) oxygen vacancy.

To further investigate the reactive oxides and oxygen vacancies generated in N-TiO_2_-0.05 under irradiation, EPR spectroscopy was employed, and the resulting EPR spectrum is shown in Figure 6. The EPR spectra of hydroxyl radical and superoxide radical generated by the sample after visible light irradiation are depicted in Figure 6a and Figure 6b, respectively. It is worth noting that no discernible free radical signal was detected in the N-TiO_2_-0.05 sample under dark conditions, suggesting that this sample did not produce free radicals without light excitation. These two free radicals were the primary reactive species responsible for N-TiO_2_-0.05 pollutant degradation [44]. The EPR spectrum of N-TiO_2_-0.05 in Figure 6c reveals the presence of oxygen vacancies, as evidenced by a distinct peak at a g value of 2.003. This observation could be attributed to the N doping, leading to the formation of Ti^3+^ complexes with oxygen from air or water. The presence of oxygen vacancies was critical in modulating the thermodynamic driving forces for photocatalytic oxidation and reduction processes. The results obtained were in agreement with the findings from the XPS analysis [45,46].

**Figure 7:**
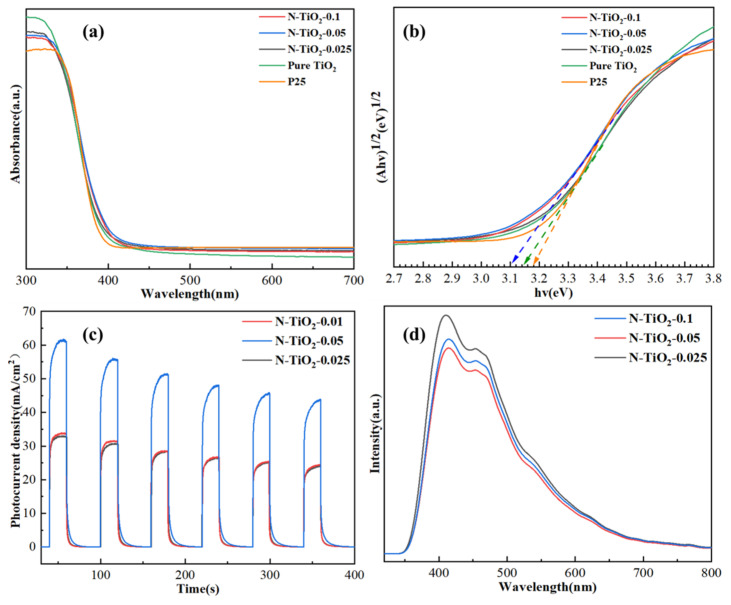
(**a**) UV–Vis diffuse reflectance spectra. (**b**) The plot of (αhν)^1/2^ versus hν. (**c**) Photoluminescence spectra. (**d**) Transient photocurrent response spectra of photocatalysts.

The optical absorption properties of N-TiO_2_-X and P25 NP were evaluated through UV-Vis diffuse reflectance analysis, enabling the measurement of their absorbance characteristics. Figure 7a,b present the corresponding diffuse reflectance UV-Vis absorption spectra and Touc plots for these samples, respectively [47]. The results demonstrate an enhanced absorption value of N-doped TiO_2_ in the range of 300~700 nm, compared with P25 NP and reference TiO_2_. Furthermore, N-TiO_2_-X exhibited a redshift in the initiation of absorption edge when compared with P25 NP. The band gap energies were calculated as 3.10, 3.14, and 3.18 eV for N-TiO_2_-0.05, reference TiO_2_, and P25 NP, respectively. Interestingly, the presence of nitrogen in the TiO_2_ lattice significantly reduced the band gap to a value close to 3.10 eV, indicating the introduction of substitutional nitrogen. This elucidated the enhanced absorption edge onset and narrower band gap observed in N-TiO_2_-0.05 compared with the reference TiO_2_ and P25 NP [36].

The efficiency of the catalyst’s electron-hole pair migration and transfer was investigated through photoluminescence (PL) emission spectroscopy. The fluorescence spectra in Figure 7c demonstrate that when excited at 320 nm, all synthesized materials exhibited prominent fluorescence peaks at 410 and 454 nm. These peaks corresponded to self-trapped excitons resulting from band gap transitions and electrons trapped by oxygen vacancies. The PL spectrum revealed a decrease in the separation efficiency of electron-hole pairs with an increase in the intensity of the PL emission peak. Among them, the fluorescence intensity of N-TiO_2_-0.05 was relatively low, indicating its low photogenerated carrier recombination rate, which favored the enhancement of its photocatalytic degradation performance [44].

The transient photocurrent responses of different samples are shown in Figure 7d. As extensively reported in the literature, a higher photocurrent density typically indicates more efficient light harvesting and separation of photogenerated charge carriers in the catalyst, which in turn enhances the overall charge carrier separation efficiency. During a 50 s irradiation period, all samples exhibited a rapid and consistent transient photocurrent response. The ranking of the photocurrent intensity could be arranged as follows: N-TiO_2_-0.05 > N-TiO_2_-0.1 > N-TiO_2_-0.025. The current density of N-TiO_2_-0.05 could reach 63 μA/cm^2^. This finding suggested that N-TiO_2_-0.05 exhibited enhanced capability in facilitating efficient transfer and separation of photogenerated carriers, thus leading to improved photocatalytic performance [48].

**Figure 8:**
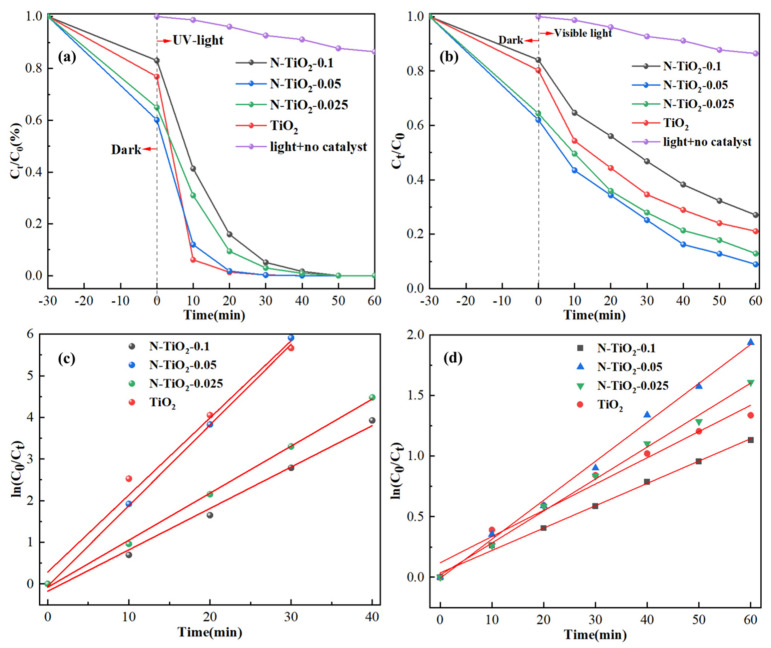
The photocatalytic degradation efficiency of the sample for TC: (**a**) photodegradation efficiency of UV light, (**b**) photodegradation efficiency of visible light, (**c**) reaction kinetics of UV light, and (**d**) reaction kinetics of visible light.

The photocatalytic activities of N-TiO_2_-X and reference TiO_2_ were systematically investigated by evaluating the photocatalytic degradation of TC under both UV and visible light irradiations, as illustrated in Figure 8. In the absence of a catalyst, the concentration of TC remained relatively stable over time under ultraviolet irradiation, as depicted in Figure 8a. This observation suggested that the photolysis of TC was negligible in the absence of TiO_2_. Furthermore, prior to light source irradiation, these synthesized TiO_2_ samples were subjected to a 30 min dark adsorption period in order to achieve adsorption equilibrium. Among them, the adsorption rate of N-TiO_2_-0.05 could reach 40%, which was primarily attributed to its substantially larger specific surface area (218 cm^3^/g) [49]. Moreover, when under UV light irradiation, N-TiO_2_-0.05 exhibited the most rapid and thorough degradation, resulting in complete removal of TC within a 30 min timeframe. The TC degradation rate of N-TiO_2_-0.025 and N-TiO_2_-0.1 reached nearly 100% within a duration of 40 min.

The potential application of catalysts in visible light-driven TC degradation was systematically investigated, as shown in Figure 8b. Under visible light irradiation (λ > 420 nm), the TC concentration remained constant in the absence of any catalyst throughout the entire irradiation period. Notably, the N-TiO_2_-0.05 catalyst exhibited an outstanding degradation efficiency of 91% for TC after 60 min of visible light exposure, surpassing that of N-TiO_2_-0.025 (73%), N-TiO_2_-0.1 (87%), reference TiO_2_ (79%), and P25 (29%) [19]. The enhanced photocatalytic performance of N-TiO_2_-0.05 in visible light could be attributed to its intense absorption and narrow band gap energy in the visible region, as elucidated in the previous UV-Vis analysis. Thus, these phenomena were all attributable to the incorporation of N atoms into the TiO_2_ structure. This suggested the existence of an optimal N-doped concentration for TiO_2_. The introduction of heavy N doping has been reported to result in an excessive number of defects, thereby diminishing the efficiency of the photocatalytic reaction. Therefore, the N-doped concentration exerts a significant influence on the catalytic performance, and appropriate doping concentration can substantially enhance the photocatalytic efficiency.

The kinetic analysis results of TC degradation under UV and visible light irradiation are shown in Figure 8c and Figure 8d, respectively. The results indicated that the pseudothermal decomposition process of TC induced by samples exhibited pseudo-first-order kinetic behavior. The rate constant of N-TiO_2_-0.05 under UV light conditions was found to be 0.194 min^−1^, which exhibited a significant enhancement compared with N-TiO_2_-0.025 (0.113 min^−1^) and N-TiO_2_-0.1 (0.099 min^−1^), by factors of 1.7 and 2, respectively. Under visible light irradiation, N-TiO_2_-0.05 exhibited a K value of 0.032 min^−1^, which was significantly higher than those of N-TiO_2_-0.025 (0.026 min^−1^), N-TiO_2_-0.1 (0.018 min^−1^), and reference TiO_2_ (0.022 min^−1^). This finding suggested that the photocatalytic activity of N-TiO_2_-0.05 was markedly enhanced relative to other samples, thereby confirming the optimal nitrogen doping concentration for achieving superior performance under visible light conditions. The rate constants for the pseudo-first-order kinetics of different samples are presented in Table S3.

The degradation of TC by N-doped TiO_2_ catalysts is summarized in Table S4. It can be observed from the table that our prepared N-doped TiO_2_ exhibited exceptional degradation performance, effectively removing TC compared with various previous photocatalysts.

To further investigate the mechanism of nitrogen-doped TiO_2_, DFT was employed to calculate the changes in band structure and density of states (DOS) induced by N doped at substitutional sites and interstitial sites in TiO_2_. In this study, a 2 × 1 × 1 supercell model of anatase TiO_2_ was initially constructed. Based on this model, the substitution doping involved replacing one oxygen atom with one nitrogen atom (labeled N_s_-doped). Similarly, interstitial doping occurred when one nitrogen atom was embedded in the interstitial position of the unit cell (labeled N_i_-doped), as shown in Figure S3 [50].

All calculations were performed using ultrasoft pseudopotentials within the generalized gradient approximation (GGA) as implemented by the Perdew–Wang 91 functional in the Cambridge Sequential Total Energy Package (CASTEP) code in Materials Studio 3.2. A Monkhorst–Pack k-point sampling of 3 × 7 × 3 was employed, along with a plane wave cutoff energy of 380 eV [50].

**Figure 9:**
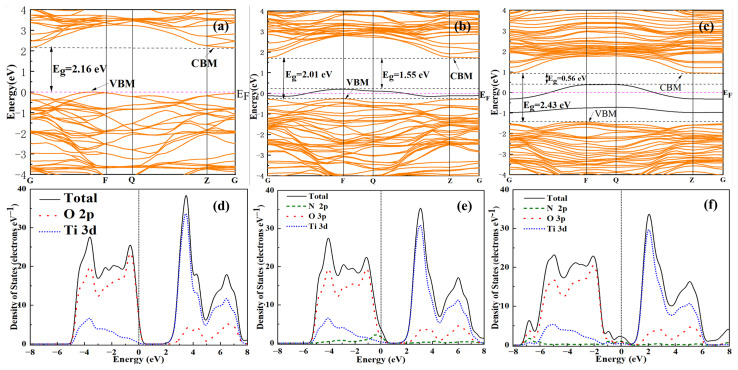
Calculated band structure of (**a**) pure TiO_2_, (**b**) N_s_ doping, and (**c**) N_i_ doping (the black line represents the impurity energy levels). The DOS and PDOS of (**d**) pure TiO_2_, (**e**) N_s_ doping, and (**f**) N_i_ doping.

The band structures of anatase TiO_2_, as well as N_s_ and N_i_ doped TiO_2_, are systematically illustrated in Figure 9a–c. The calculated bandgap of pure anatase TiO_2_ was determined to be 2.16 eV (Figure 9a), which exhibited excellent consistency with previously reported theoretical data [51,52]. However, this value underestimated the experimental band gap (E_g_ = 3.23 eV) due to the limitations of DFT. Specifically, DFT did not account for the discontinuity in the exchange-correlation potential [50]. Regardless of whether they were N_s_-doped or N_i_-doped, the Fermi levels (E_F_) crossed above their respective impurity energy levels, indicating that these states were not fully occupied by electrons in the ground state. In the N_s_-doped system, the impurity energy level was slightly elevated above the valence band maximum (VBM), functioning as a shallow acceptor. This configuration resulted in a reduced calculated band gap of 2.01 eV (Figure 9b). In contrast, the N_i_-doped system exhibited two isolated impurity levels positioned significantly higher than the VBM. Furthermore, both the VBM and conduction band minimum (CBM) underwent downward shifts toward lower energy levels, collectively contributing to an enlarged band gap of 2.43 eV in the calculated results (Figure 9c). While this increase in the band gap was not favorable for visible light absorption, considering the influence of impurity energy levels, the visible light absorption capability of the N_i_-doped system was actually enhanced [50]. In this study, due to the modest reduction in the band gap of N-doped TiO_2_ and in conjunction with DFT calculations, it was reasonable to infer that the N-doped TiO_2_ exhibited N_s_ doping. This conclusion aligned with the findings from XPS analysis.

To investigate the influence of nitrogen doping on the electronic structures of TiO_2_, the calculated total density of states (DOS) and projected density of states (PDOS) for pristine TiO_2_, N_s_-doped TiO_2_, and N_i_-doped TiO_2_ are presented in Figure 9d–f. These spectral distributions offer a clear visualization of dopant-induced modifications to the electronic band structures, facilitating a systematic analysis of doping effects across various substitutional impurities. Specifically, the PDOS profiles elucidate orbital-specific contributions, providing deeper insights into charge carrier dynamics and defect state formation mechanisms within the doped systems. The valence band (VB) of pure TiO_2_ was primarily composed of O 2p states with a minor contribution from Ti 3d states, as shown in Figure 9d. In contrast, the conduction band (CB) exhibited a dominant Ti 3d character with minor admixture of O 2p states. The VB maximum was primarily governed by O 2p orbitals, while the CB minimum was determined by Ti 3d states, consistent with the electronic structure characteristics of TiO_2_ semiconductor materials [51]. This distribution of electronic states suggested that the O-Ti bond in anatase TiO_2_ was primarily ionic, with some weak covalent character [53]. By comparing Figure 9e,f, it is evident that the N 2P state’s contribution to the CB of TiO_2_ was minimal, thereby resulting in a negligible impact of N doping on the CB [54]. The peak of the N 2p states was observed at the VB top upon N_s_ doping, whereas it was located at the VB bottom with N_i_ doping [55].

In summary, based on DFT calculations, N_s_ doping could narrow the energy band of TiO_2_, whereas N_i_ doping introduced two impurity energy levels, enhancing the material’s ability to absorb visible light. Combined with DOS and PDOS analyses, the N 2p states predominantly contributed to the VB of TiO_2_, irrespective of whether the material was N_s_ doping or N_i_ doping.

**Figure 10:**
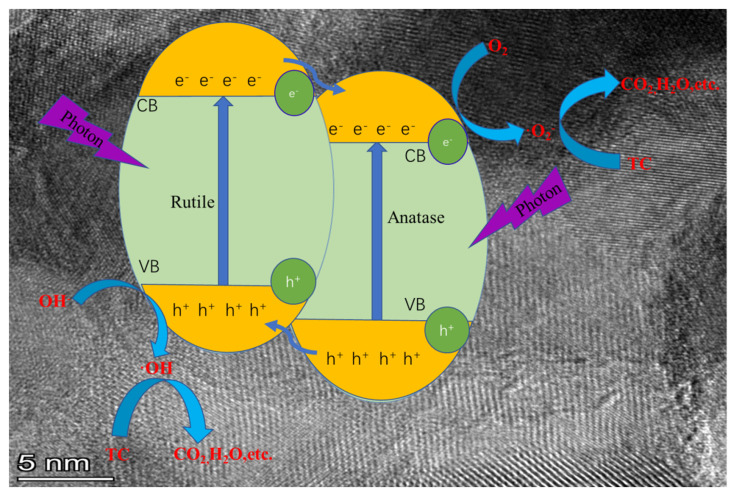
The mechanism of photocatalytic degradation of TC under visible light by hierarchical micro/nanostructure N-TiO_2_ photocatalysts.

Based on the comprehensive analysis of XRD, XPS, EPR spectroscopy, and DFT calculations, corroborated by previous literature reports, a plausible reaction mechanism for the visible light photocatalytic performance of nitrogen-doped TiO_2_ was proposed (Figure 10). The integrated experimental and theoretical studies unequivocally demonstrated that nitrogen doping into the TiO_2_ lattice significantly modified its electronic band structure. Specifically, this doping strategy introduced intermediate states derived from nitrogen 2p orbitals, which were situated just above the oxygen 2p valence band maximum. This structural modification not only narrowed the bandgap, thereby enhancing visible light absorption, but also created favorable energetic conditions for efficient charge carrier separation and transfer processes [34,55,56]. The N-TiO_2_ materials generated a substantial amount of photoexcited electron-hole pairs (e^−^/h^+^) under visible light irradiation. Moreover, the heterojunction phase composition in the N-TiO_2_ system effectively separated the photogenerated e^−^/h^+^ pairs. Due to the high electron affinity of anatase, photoexcited electrons could more readily flow from rutile to anatase, leading to more efficient separation of e^−^/h^+^ pairs. The electrons reduced the adsorbed O_2_ to superoxide ions (·O_2_^−^), whereas the holes reacted with OH^−^ groups to generate hydroxyl radicals, which were responsible for the mineralization of TC under visible light irradiation. These results demonstrated that the N-TiO_2_-0.05 catalyst exhibited remarkable photocatalytic efficiency under visible light illumination, which could be predominantly ascribed to the synergistic effect of the optimized nitrogen doping concentration and the formation of a hierarchical micro/nanostructured heterojunction architecture.

## 4. Conclusions

We report a facile one-step solvothermal synthesis method for nitrogen-doped TiO_2_ nanoparticles (N-TiO_2_), which exhibited hierarchical micro/nanostructures and a significantly increased surface area. The synthesized N-TiO_2_ nanoparticles showed enhanced visible light absorption and efficient separation of photogenerated electron-hole pairs. Furthermore, they demonstrated exceptional photocatalytic activity in the degradation of antibiotics under both UV and visible light irradiation.

Structural characterization confirmed that the N-doped TiO_2_ predominantly consisted of anatase phase with a minor rutile phase, as evidenced by XRD analysis. The SEM images revealed that the spherical diameter of TiO_2_ nanoparticles increased from 200 to 250 nm with higher N/Ti molar ratios. Importantly, nitrogen doping markedly enhanced the surface area of TiO_2_, increasing it 3.8-fold from 57 to 218 cm^3^/g in N-TiO_2_-0.05 compared with pure TiO_2_. Various analytical techniques, including XPS and EDS mapping, confirmed the incorporation of nitrogen into the TiO_2_ lattice. The red shift in the light absorption edge further supported the structural modification induced by nitrogen doping.

The photocatalytic performance of N-TiO_2_ was outstanding, achieving nearly 100% degradation of antibiotics under UV light and 87% under visible light, surpassing that of undoped TiO_2_. This superior performance could be attributed to the optimal nitrogen doping level and the hierarchical micro/nanostructures, which effectively enhanced the surface area and reduced the band gap. These findings provided a solid experimental and theoretical basis for advancing photocatalytic applications in antibiotic degradation using N-doped TiO_2_ nanoparticles.

## Data Availability

The raw data supporting the conclusions of this article will be made available by the authors on request.

## Supplementary Materials

The following supporting information can be downloaded at https://www.mdpi.com/article/10.3390/ma18102400/s1. Table S1. The unit cell parameters of anatase TiO_2_ and rutile TiO_2_; Table S2. Specific surface areas, pore volume, and pore diameter of samples; Table S3. The pseudo-first-order kinetic rate constants of different samples; Table S4. Comparison of the removal efficiency of various photocatalysts for TC; Figure S1. A (101) and R (110) peaks slightly shift to higher angles; Figure S2. Element mapping of N-TiO_2_-0.5; Figure S3. The 2 × 2 × 1 supercell model considered in this work. (a) A primitive unit cell of anatase TiO_2_, (b) N_s_ doped, and (c) N_i_ doped. The red, gray, and blue spheres represent the O, Ti, and N atoms, respectively. References [28,43,48,57,58,59,60] are cited in the Supplementary Materials.
