# Supplementary material for: One-Step Synthesis of Nitrogen-Doped TiO2 Heterojunctions and Their Visible Light Catalytic Applications
Source: Materials (Basel). 2025 May 21;18(10):2400. doi: 10.3390/ma18102400 (PMC12113483; doi:10.3390/ma18102400)
Supplement: Supplementary file 1 [file materials-18-02400-s001.zip › materials-3591066-supplementary.pdf]

Supplementary Material

One-step synthesis of nitrogen-doped TiO_2 heterojunction and their visible light catalytic applications

Peng Lian [a,b], Aimiao Qin [a,*], Zhisen Liu[b], Hao Ma[b], Lei Liao [a] and Kaiyou Zhang [a], Yingxi Qin[a]

[a] Key Lab New Processing Technology for Nonferrous Metals & Materials Ministry of Education, Guangxi Key Laboratory of Optical and Electronic Materials and Devices, Guangxi Key Laboratory of Environmental Pollution Control Theory and Technology, College of Materials science & engineering, Guilin University of Technology, Guilin 541004, China
[b] Guangdong University of Petrochemical Technology, Maoming, Guangdong ,525000, China
* Corresponding authors: *2005032@glut.edu.cn (A. M. Qin).*

Table S1. The unit cell parameters of anatase TiO_2 and rutile TiO_2.

**Samples**	**Anatase TiO_2**			**Rutile TiO_2**		
	**a**	**b**	**c**	**a**	**b**	**c**
N-TiO_2-0.025	3.7845	3.7845	9.5605	4.5949	4.5949	2.9623
N-TiO_2-0.05	3.7842	3.7842	9.5014	4.5931	4.5931	2.9589
N-TiO_2-0.1	3.7834	3.7834	9.4999	4.5888	4.5888	2.9555
Tetragonal crystal system, α=β=γ=90°						

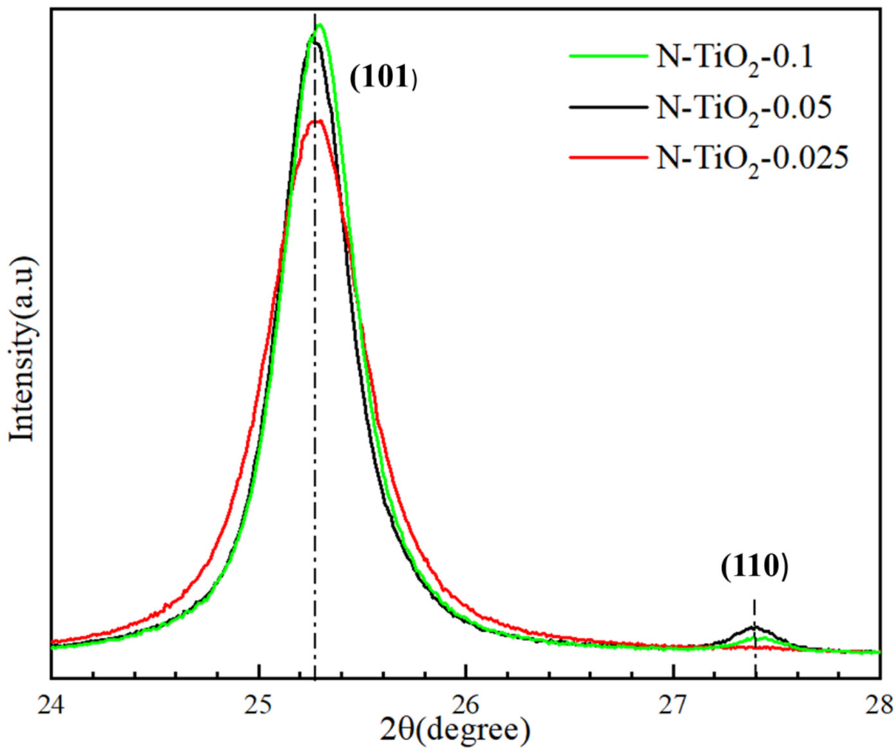

Figure S1. A (101) and R (110) peaks slightly shift to higher angles.

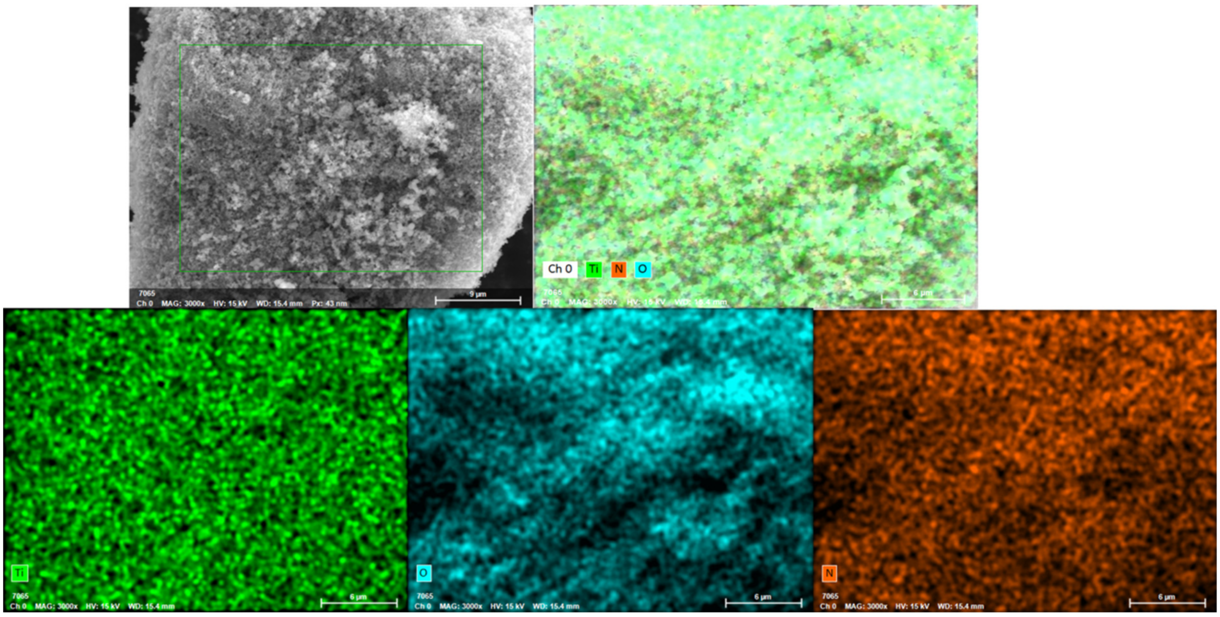

Figure S2. Element mapping of N-TiO_2-0.5.

Table S2. Specific surface areas, pore volume, and pore diameter of samples.

Samples	Surface Area(m^2/g)	BJH Pore Volume(cm^3/g)	BJH Average Pore Size(nm)
N-TiO_2-0.025	124	0.27	12.63
N-TiO_2-0.05	218	0.73	8.51
N-TiO_2-0.1	55	0.24	7.39
TiO_2	57	0.18	9.07

Table S3. The pseudo-first-order kinetic rate constants of different samples.

Samples	UV Light		Visible Light	
	Kinetic rate constants (k, min^{-1})	Correlation coefficients (R^2)	Kinetic rate constants (k, min^{-1})	Correlation coefficients (R^2)
N-TiO_2-0.025	0.1130	0.9882	0.02641	0.9957
N-TiO_2-0.05	0.1937	0.9924	0.03217	0.9953
N-TiO_2-0.1	0.0994	0.9983	0.01846	0.9960
TiO_2	0.1852	0.9845	0.02167	0.9761

Table S4. Comparison of the removal efficiency of various photocatalysts for TC.

Photocatalyst	light type	Light time (min)	Degradation rate (%)	References
N-TiO_2	visible light	60	92	[1]
N-TiO_2	visible light	120	74	[2]
TiO_2@N-HCS/GCN	visible light	120	85%	[3]
N-TiO_2	visible light	60	79	[4]
N-TiO_2-Bi_2WO_6	visible light	60	87.2	[5]
N-TiO_2	visible light	120	74.4	[6]
N-TiO_2	visible light	60	90	[7]
N-TiO_2	UV/visible light	60	Nearly100/87	This work

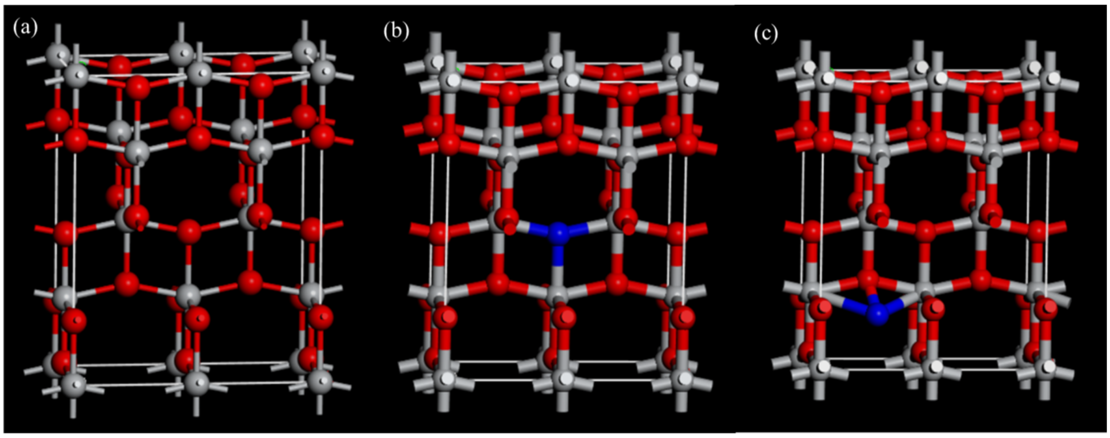

Figure S3. The 2 × 2 × 1 supercell model considered in this work(a) A primitive unit cell of anatase TiO2, (b)N_s doped, (c) N_i doped. The red, gray, and blue spheres represent the O, Ti, and N atoms, respectively.

References

[1]S. Sun, M. Sun, W. Ming, Y. Ma, H. LiG. Xu,Amorphous titanium dioxide with synergistic effect of nitrogen doping and oxygen vacancies by photoexcited sol-gel preparation for enhanced photodegradation of tetracycline,J. Photochem. Photobiol. A: Chem. 461（2025）https://doi.org/10.1016/j.jphotochem.2024.116165.

[2]F.T. Tao, C. Hu, J. C. S. Wu, V.H. NguyenK.L. Tung,Influence of nitrogen sources on N-doped reduced TiO_2 prepared using atmospheric plasma spraying for photocatalytic tetracycline and ciprofloxacin degradation,Sep. Purif. Technol. 326（2023）https://doi.org/10.1016/j.seppur.2023.124784.

[3]A. B. DehkordiA. Badiei,Insight into the activity of TiO_2@nitrogen-doped hollow carbon spheres supported on g-C_3N_4 for robust photocatalytic performance,Chemosphere 288（2022）https://doi.org/10.1016/j.chemosphere.2021.132392.

[4]X. Tang, Z. WangY. Wang,Visible active N-doped TiO_2/reduced graphene oxide for the degradation of tetracycline hydrochloride,Chemical Physics Letters 691（2018）408-414,https://doi.org/10.1016/j.cplett.2017.11.037.

[5]Y. Tang, T. Li, W. Xiao, Z. Huang, H. Wen, W. SituX. Song,Degradation mechanism and pathway of tetracycline in milk by heterojunction N-TiO_2-Bi_2WO_6 film under visible light,Food Chem. 401（2023）134082,https://doi.org/10.1016/j.foodchem.2022.134082.

[6]Y. Zhou, T. Cai, S. Liu, Y. Liu, H. Chen, Z. Li, J. Du, Z. Lei,N-doped magnetic three-dimensional carbon microspheres@TiO_2 with a porous architecture for enhanced degradation of tetracycline and methyl orange via adsorption/photocatalysis synergy,Chem. Eng. J. 411（2021）https://doi.org/10.1016/j.cej.2021.128615.

[7]S. Li, C. Jiang, Y. Zhang, J. Tian, H. Yang, C. Wang, Synergistic effect of N doping and oxygen vacancies over TiO_2 nanosheets with enhanced photocatalytic removal of tetracycline,Catal. Today 440（2024）https://doi.org/10.1016/j.cattod.2024.114830.
